# New Mode of Reducing Strangulated Hernia

**Published:** 1852-09

**Authors:** 


*New Mode of Reducing Strangulated Hernia.*—Dr. Wise makes the following interesting communication in a letter addressed to Professor Syme:—

"The following are the particulars I promised to send you, regarding a new method of reducing strangulated hernia. While I had charge of an hospital in India, an elderly man was brought to it with a strangulated inguinal hernia. After in vain employing the usual means of reduction, I was preparing to liberate the gut with a knife, when a Mussulman gentleman suggested that the following method should be first tried, as he had seen it successful. As it appeared most simple and effective, I at once proceeded to try it. The patient was placed upon a table, and a long sheet, folded several times on itself, was carried round the lower part of the abdomen of the patient, was twisted on itself in front, and again on the sides, so as to enable an assistant, standing on each side of the patient, to hold the extremities of the sheet, and to pull them gently upwards, or towards the patient's head, while a third assistant held the feet steady, and the surgeon used the taxis.

"As the gut immediately above the strangulated portion was superficial and distended with air and liquid, it was drawn upwards with considerable force from the hernial sac, which was assisted by the surgeon using the taxis; when the strangulated portion was immediately reduced.

"This simple method may, in a very large proportion of cases, be employed with perfect safety and at an early period, before inflammation and thickening has complicated and increased so much the danger of the operation, which is thus rendered unnecessary."—*London Journal of Medicine.*

